# Supplementary material for: Early Fever in Allogeneic Stem Cell Transplantation: Are Presepsin and YKL-40 Valuable Diagnostic Tools?
Source: J Clin Med. 2024 Oct 8;13(19):5991. doi: 10.3390/jcm13195991 (PMC11478026; doi:10.3390/jcm13195991)
Supplement: Supplementary file 1 [file jcm-13-05991-s001.zip › jcm-3218169-supplementary.pdf]

Supplementary material

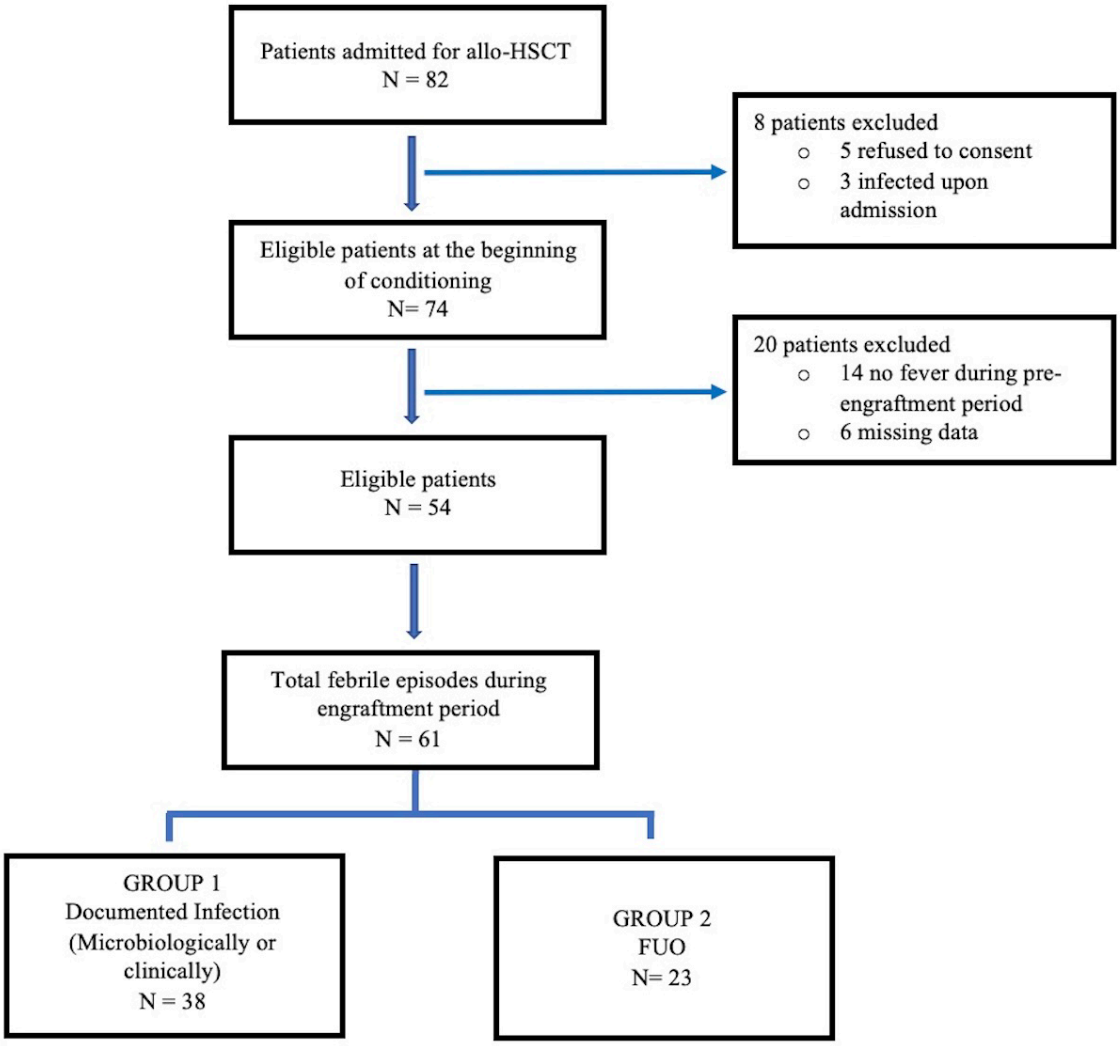

Supplementary Figure S1. Flowchart of the study population

Allo-HSCT, allogeneic hematopoietic stem cell transplantation; FUO, fever of unknown origin

	Biomarker levels	Friedman test p-value	Post-hoc comparison, adjusted p-value
**PSP (ng/mL) 4)**			
Day 0	1.442 (0.560 – 2.424)		Day 0 vs Day 1 (p < 0.0001) ****<br>Day 0 vs Day 3 (p < 0.0001) ****<br>Day 0 vs Day 5 (p = 0.003) **<br>Day 0 vs Day 7 (p = 0.123)
Day 1	4.835 (1.979 – 15.60)		Day 1 vs Day 3 (p > 0.1)<br>Day 1 vs Day 5 (p = 0.033) *<br>Day 1 vs Day 7 (p = 0.0004) ***
Day 3	5.160 (2.492 – 10.110)		Day 3 vs Day 5 (p = 0.052)<br>Day 3 vs Day 7 (p = 0.0008) ***
Day 5	2.680 (1.553 – 6.489)		Day 5 vs Day 7 (p > 0.1)
Day 7	2.207 (1.524 – 4.206)	p < 0.0001	
**YKL-40 (ng/mL)**			
Day 0	37.60 (21.80 – 70.65)		Day 0 vs Day 1 (p<0.0001) ****<br>Day 0 vs Day 3 (p<0.0001) ****<br>Day 0 vs Day 5 (p = 0.697)<br>Day 0 vs Day 7 (p = 0.323)
Day 1	87.10 (49.40 – 167.60)		Day 1 vs Day 3 (p > 0.1)<br>Day 1 vs Day 5 (p = 0.046) *<br>Day 1 vs Day 7 (p = 0.123)
Day 3	91.80 (50.65 - 155.30)		Day 3 vs Day 5 (p = 0.026) *<br>Day 3 vs Day 7 (p = 0.073)
Day 5	60.80 (31.60 – 118.70)		Day 5 vs Day 7 (p > 0.1)
Day 7	59.40 (36.18 – 133.70)	p < 0.0001	
**PCT (µg/L)**			
Day 0	N/D		Day 0 vs Day 1 (p<0.0001) ****<br>Day 0 vs Day 3 (p<0.0001) ****<br>Day 0 vs Day 5 (p<0.0001) ****<br>Day 0 vs Day 7 (p=0.029) *
Day 1	0.46 (0.138 – 4.53)		Day 1 vs Day 3 (p > 0.1)<br>Day 1 vs Day 5 (p = 0.250)<br>Day 1 vs Day 7 (p < 0.0001) ****
Day 3	0.45 (0.22 – 1.09)		Day 3 vs Day 5 (p = 0.151)<br>Day 3 vs Day 7 (p < 0.0001) ****
Day 5	0.23 (0.15 – 0.47)		Day 5 vs Day 7 (p = 0.058)
Day 7	0.19 (0.06 – 0.30)	p < 0.0001	

Supplementary Table S1. Differences in PSP, YKL-40 and PCT median concentrations in the infection group across Day 0, 1, 3, 5 and 7 with post-hoc pairwise comparisons following a significant Friedman test result (Dunn's test with Bonferroni correction). All values in both groups for PCT Day 0 were bellow detection limit (<0.6 µg/L). To ensure a conservative estimate and clinical relevance, for statistical analysis values were substituted with the detection limit 0.6 µg/L. * = p < 0.05, ** = p < 0.01, *** = p < 0.001, **** = p < 0.0001. N/D, nondetectable; PCT, procalcitonin; PSP, presepsin

	Biomarker levels	Friedman test p-value	Post-hoc comparison, adjusted p-value
**PSP (ng/mL) 4)**			
Day 0	1.220 (0.762 – 1.560)		Day 0 vs Day 1 (p < 0.0001) ****<br>Day 0 vs Day 3 (p = 0.013)<br>Day 0 vs Day 5 (p = 0.090)<br>Day 0 vs Day 7 (p = 0.502)
Day 1	2.060 (1.322 – 5.383)		Day 1 vs Day 3 (p > 0.1)<br>Day 1 vs Day 5 (p = 0.284)<br>Day 1 vs Day 7 (p=0.0445) *
Day 3	2.403 (1.236 – 5.686)		Day 3 vs Day 5 (p > 0.1)<br>Day 3 vs Day 7 (p > 0.1)
Day 5	2.168 (1.204 – 3.596)		Day 5 vs Day 7 (p > 0.1)
Day 7	1.614 (1.320 – 3.354)	p <0.0001	
**YKL-40 (ng/mL)**			
Day 0	26.90 (21.30 – 44.90)		Day 0 vs Day 1 (p=0.001) ***<br>Day 0 vs Day 3 (p < 0.0001) ****<br>Day 0 vs Day 5 (p=0.0003) ***<br>Day 0 vs Day 7 (p=0.028) *
Day 1	70.30 (38.10 – 109.70)		Day 1 vs Day 3 (p > 0.1)<br>Day 1 vs Day 5 (p > 0.1)<br>Day 1 vs Day 7 (p > 0.1)
Day 3	86.40 (41.80 – 101.90)		Day 3 vs Day 5 (p > 0.1)<br>Day 3 vs Day 7 (p = 0.764)
Day 5	52.90 (39.00 – 98.40)		Day 5 vs Day 7 (p > 0.1)
Day 7	56.00 (29.30 – 104.70)	p <0.0001	
**PCT (μg/L)**			
Day 0	N/D		Day 0 vs Day 1 (p<0.0001) ****<br>Day 0 vs Day 3 (p<0.0001) ****<br>Day 0 vs Day 5 (p=0.003) **<br>Day 0 vs Day 7 (p > 0.1)
Day 1	0.225 (0.0975 – 1.253)		Day 1 vs Day 3 (p > 0.1)<br>Day 1 vs Day 5 (p = 0.450)<br>Day 1 vs Day 7 (p = 0.0001) ***
Day 3	0.17 (0.10 – 0.47)		Day 3 vs Day 5 (p = 0.622)<br>Day 3 vs Day 7 (p=0.0003) ***
Day 5	0.13 (0.08 – 0.26)		Day 5 vs Day 7 (p = 0.197)
Day 7	0.09 (0.06 – 0.13)	p < 0.0001	

Supplementary Table S2. Differences in PSP, YKL-40 and PCT median concentrations in FUO group across Day 0, 1, 3, 5 and 7 with post-hoc pairwise comparisons following a significant Friedman test result (Dunn's test with Bonferroni correction). All values in both groups for PCT Day 0 were bellow detection limit (<0.6 μg/L). To ensure a conservative estimate and clinical relevance, for statistical analysis values were substituted with the detection limit 0.6 μg/L. * = p < 0.05, ** = p < 0.01, *** = p < 0.001, **** = p < 0.0001. FUO, fever of unknown origin; N/D, nondetectable PCT, procalcitonin; PSP, presepsin

Biomarker	Day 0	Day 1	Day 3	Day 5	Day 7
**PSP/YKL-40**					
r	0.36	0.51	0.36	0.06	0.28
p	0.008	< 0.001	0.005	0.652	0.029
**PSP/PCT**					
r	-0.09	0.67	0.78	0.71	0.52
p	0.532	< 0.001	< 0.001	< 0.001	< 0.001
**YKL-40/PCT**					
r	-0.19	0.45	0.38	0.26	0.36
p	0.174	< 0.001	0.002	0.05	0.004

Supplementary Table S3. Spearman correlations between serum levels of PSP, YKL-40 and PCT, independent of infection status. A strong positive correlation was defined as $r > 0.6$ and $p \leq 0.05$. PCT, procalcitonin; PSP, presepsin; r, Spearman correlation coefficient

**Biomarker**	**OR**	**95% CI**	**R^2**	**p value**
**Day1**				
Day 1 PSP > 2.910 ng/mL	4.06	1.39 – 12.70	0.1032	0.012**
Day 1 YKL > 111.40 ng/mL	2.91	0.94 – 10.30	0.0545	0.075
Day 1 PCT > 0.57 µg/L	2.55	0.85 – 8.37	0.0448	0.104*
**Day 3**				
Day 3 PSP > 3.144 ng/mL	5.61	1.87 – 18.38	0.1479	0.003**
Day 3 YKL > 108.40 ng/mL	3.46	1.05 – 13.68	0.0666	0.053*
Day 3 PCT > 0.28 µg/L	7.93	2.56 – 27.66	0.1993	0.0006**

Supplementary Table S4. Results of the univariate binary logistic regression analysis of PSP, YKL-40 and PCT as predictors of infection on Day 1 and Day 3 after the onset of FE. Analysis was done using dichotomized biomarker values (higher or lower than cutoff).

Cox-Snell's R^2, ** significant predictors $p < 0.05$, *borderline predictors $0.05 < p < 0.10$

FE, febrile episode; OR, odds ratio; PCT, procalcitonin; PSP, presepsin; R^2, coefficient of determination;

Predictor	OR	95% CI	p	VIF
**Day 1 Model 1: R^2=0.1605, AUC=0.703, p = 0.054**				
PSP > 2.910 ng/mL	2.965	0.876 - 10.810	0.086	1.36
YKL-40 > 111.40 ng/mL	1.543	0.391 - 6.271	0.532	1.35
PCT > 0.57 µg/L	1.513	0.424 – 5.506	0.520	1.24
**Day 1 Model 2: R^2=0.1528, AUC=0.689, p = 0.027***				
PSP > 2.910 ng/mL	3.463	1.092 – 11.520	0.038*	1.17
PCT > 0.57 µg/L	1.638	0.474 – 5.826	0.433	1.17
**Day 1 Model 3: R^2=0.1617, AUC=0.695 p = 0.052**				
PSP > 2.910 ng/mL	4.201	1.167 – 17.830	0.036*	1.38
PCT > 0.57 µg/L	1.951	0.512 – 7.997	0.332	1.39
ATG	0.574	0.102 – 2.869	0.505	1.59
**Day 3 Model 4: R^2=0.3419, AUC=0.796, p = 0.0005*****				
PSP > 3.144 ng/mL	2.517	0.652 – 9.620	0.172	1.42
YKL-40 > 108.40 ng/mL	2.593	0.666 – 11.650	0.183	1.06
PCT > 0.28 µg/L	4.734	1.277 – 18.970	0.022*	1.42
**Day 3 Model 5: R^2=0.3102, AUC=0.771, p = 0.0004*****				
PSP > 3.144 ng/mL	2.713	0.721 – 10.150	0.133	1.40
PCT > 0.28 µg/L	4.998	1.380 – 19.610	0.016*	1.40
**Day 3 Model 6: R^2=0.3504, AUC=0.796, p = 0.0004*****				
PSP > 3.144 ng/mL	3.717	0.893 – 17.200	0.075	1.47
PCT > 0.28 µg/L	9.391	1.963 – 69.680	0.010*	1.61
ATG	0.236	0.026 – 1.448	0.148	1.41

Supplementary Table S5. Results of the multivariable binary logistic regression analysis of different biomarker combinations as predictors of infection on Day 1 and Day 3 after the onset of FE.

Nagelkerke's R squared, p value by likelihood ratio test. AUC, area under the ROC curve; CI, confidence interval; FE, febrile episode; OR, odds ratio; PCT, procalcitonin; PSP, presepsin; R^2, coefficient of determination; VIF, variance inflation factor

	Infection (n=30)	FUO (n=14)	p-value
**PSP (ng/mL)**			
Day 1	5.250 (2.264 – 16.180)	1.990 (1.308 – 4.743)	0.025*
Day 3	5.264 (3.220 – 10.410)	1.849 (1.60 – 3.693)	0.001**
Day 5	2.939 (1.727 – 6.489)	1.480 (1.112 – 3.369)	0.012*
Day 7	2.213 (1.724 – 3.453)	1.472 (1.235 – 2.784)	0.044*
**YKL-40 (ng/mL)**			
Day 1	87.10 (49.10 – 146.10)	72.75 (28.80 – 106.30)	0.180
Day 3	89.65 (50.65 – 99.65)	67.25 (32.38 – 100.9)	0.186
Day 5	58.95 (31.28 – 118.70)	58.15 (35.98 – 97.13)	0.906
Day 7	58.45 (33.48 – 129.20)	54.75 (32.30 – 93.15)	0.770
**PCT (μg/L)**			
Day 1	0.22 (0.11 – 1.35)	0.10 (0.08 – 0.33)	0.058
Day 3	0.45 (0.23 – 1.03)	0.17 (0.10 – 0.19)	0.0012**
Day 5	0.23 (0.15 – 0.55)	0.13 (0.08 – 0.18)	0.014*
Day 7	0.20 (0.08 – 0.31)	0.08 (0.06 – 0.11)	0.008 **

Supplementary Table S6. Comparison of biomarker concentrations in neutropenic patients between infection and FUO group. The data are expressed as medians and interquartile ranges. Differences in biomarker values between the infection and FUO group were tested using the Mann-Whitney U test. * = $p < 0.05$, ** = $p < 0.01$. FUO, fever of unknown origin; PCT, procalcitonin; PSP, presepsin

	Infection (n=8)	FUO (n=9)	p-value
**PSP (ng/mL)**			
Day 1	8.745 (5.719 – 17.750)	2.690 (1.293 – 12.150)	0.059
Day 3	5.899 (4.441 – 12.880)	2.622 (2.032 – 9.359)	0.075
Day 5	3.522 (2.065 – 4.568)	2.628 (1.582 – 4.224)	0.481
Day 7	2.099 (1.924 – 2.966)	2.210 (0.988 – 3-750)	0.963
**YKL-40 (ng/mL)**			
Day 1	111.50 (41.75 – 198.90)	67.20 (38.85 – 143.00)	0.673
Day 3	74.90 (47.15 – 166.40)	92.80 (46.80 – 125.20)	0.963
Day 5	37.65 (21.93 – 132.10)	52.90 (40.85 – 169.90)	0.371
Day 7	55.85 (22.83 – 184.10)	61.80 (21.94 – 113.40)	0.673
**PCT (µg/L)**			
Day 1	2.750 (0.290 – 5.958)	0.240 (0.145 – 3.600)	0.174
Day 3	0.685 (0.443 – 2.025)	0.260 (0.105 – 1.400)	0.174
Day 5	0.420 (0.165 – 0.750)	0.200 (0.075 – 0.415)	0.166
Day 7	0.215 (0.105 – 0.348)	0.090 (0.060 – 0.225)	0.115

Supplementary Table S7. Comparison of biomarker concentrations in non-neutropenic patients between infection and FUO group. The data are expressed as medians and interquartile ranges. Differences in biomarker values between the infection and FUO group were tested using the Mann-Whitney U test. * = $p < 0.05$, ** = $p < 0.01$. FUO, fever of unknown origin; PCT, procalcitonin; PSP, presepsin

Biomarker	Time point (Day)	MDRO + (n=11)	MDRO - (n=16)	p-value
PSP (ng/mL)	Day 1	3.232 (1.460 – 5.250)	3.894 (1.698 – 8.850)	0.577
	Day 3	5.160 (2.106 – 10.100)	4.533 (2.147 – 7.163)	0.636
	Day 5	2.680 (0.688 – 9.766)	2.186 (1.409 -4.003)	0.707
	Day 7	2.218 (0.597 – 8.215)	2.007 (1.355 – 4.198)	0.904
YKL-40 (ng/mL)	Day 1	81.10 (49.40 – 162.70)	87.10 (50.33 – 138.20)	0.991
	Day 3	91.80 (72.80 – 182.80)	74.90 (44.73 – 115.00)	0.144
	Day 5	60.80 (31.70 – 90.80)	52.30 (26.10 – 91.00)	0.725
	Day 7	55.80 (36.80 – 128.20)	57.30 (23.15 – 100.90)	0.707
PCT (µg/L)	Day 1	0.86 (0.11 – 6.00)	0.18 (0.12 – 3.43)	0.535
	Day 3	0.45 (0.22 – 1.08)	0.36 (0.15 – 0.75)	0.504
	Day 5	0.23 (0.15 – 0.55)	0.16 (0.08 – 0.42)	0.245
	Day 7	0.15 (0.12 – 0.42)	0.08 (0.06 – 0.22)	0.127

Supplementary Table S8. Serum values of PSP, YKL-40, and PCT during the first seven days after the onset of the febrile episode (Day 1, Day 3, Day 5, Day 7) according to the type of bacteria isolated (multidrug-resistant organisms positive vs multidrug-resistant organisms negative). The data are expressed as medians and interquartile ranges. Differences in biomarker values between MDRO+ and MDRO- group were tested using the Mann-Whitney U test. * = $p < 0.05$, ** = $p < 0.01$, *** = $p < 0.001$. FUO, fever of unknown origin; MDRO+, positive to multidrug-resistant organisms; MDRO-, negative for multidrug-resistant organisms; N/A, not applicable; N/D, nondetectable; PCT, procalcitonin; PSP, presepsin
